# Impact of cryopreservation on viability, gene expression and function of enteric nervous system derived neurospheres

**DOI:** 10.3389/fcell.2023.1196472

**Published:** 2023-06-12

**Authors:** Sabine Heumüller-Klug, Kristina Maurer, María Á. Tapia-Laliena, Carsten Sticht, Anne Christmann, Handan Mörz, Rasul Khasanov, Elvira Wink, Steven Schulte, Wolfgang Greffrath, Rolf-Detlef Treede, Lucas M. Wessel, Karl-Herbert Schäfer

**Affiliations:** ^1^ Department of Pediatric Surgery, Medical Faculty Mannheim, University of Heidelberg, Mannheim, Germany; ^2^ Medical Research Centre Mannheim, Medical Faculty Mannheim, University of Heidelberg, Mannheim, Germany; ^3^ AGENS, University of Applied Sciences Kaiserslautern Campus Zweibrücken, Kaiserslautern, Germany; ^4^ Mannheim Center for Translational Neuroscience (MCTN), Department of Neurophysiology, Medical Faculty Mannheim, University of Heidelberg, Mannheim, Germany

**Keywords:** enteric neurospheres, aganglionosis, cryopreservation, cell transplantation, freezing, enteric nervous system, calcium imagaing

## Abstract

**Introduction:** Impairment of both the central and peripheral nervous system is a major cause of mortality and disability. It varies from an affection of the brain to various types of enteric dysganglionosis. Congenital enteric dysganglionosis is characterized by the local absence of intrinsic innervation due to deficits in either migration, proliferation or differentiation of neural stem cells. Despite surgery, children’s quality of life is reduced. Neural stem cell transplantation seems a promising therapeutic approach, requiring huge amounts of cells and multiple approaches to fully colonize the diseased areas completely. A combination of successful expansion and storage of neural stem cells is needed until a sufficient amount of cells is generated. This must be combined with suitable cell transplantation strategies, that cover all the area affected. Cryopreservation provides the possibility to store cells for long time, unfortunately with side effects, i.e., upon vitality.

**Methods:** In this study we investigate the impact of different freezing and thawing protocols (M1-M4) upon enteric neural stem cell survival, protein and gene expression, and cell function.

**Results:** Freezing enteric nervous system derived neurospheres (ENSdN) following slow-freezing protocols (M1-3) resulted in higher survival rates than flash-freezing (M4). RNA expression profiles were least affected by freezing protocols M1/2, whereas the protein expression of ENSdN remained unchanged after treatment with protocol M1 only. Cells treated with the most promising freezing protocol (M1, slow freezing in fetal calf serum plus 10% DMSO) were subsequently investigated using single-cell calcium imaging. Freezing of ENSdN did not alter the increase in intracellular calcium in response to a specific set of stimuli. Single cells could be assigned to functional subgroups according to response patterns and a significant shift towards cells responding to nicotine was observed after freezing.

**Discussion:** The results demonstrate that cryopreservation of ENSdN is possible with reduced viability, only slight changes in protein/gene expression patterns and without an impact on the neuronal function of different enteric nervous system cell subtypes, with the exception of a subtle upregulation of cells expressing nicotinergic acetylcholine receptors. In summary, cryopreservation presents a good method to store sufficient amounts of enteric neural stem cells without neuronal impairment, in order to enable subsequent transplantation of cells into compromised tissues.

## Introduction

The impairment of both the central and peripheral nervous system is a major cause of mortality and disability (Mathers and Loncar, 2006). It varies from affection of the brain (stroke, cerebrovascular diseases and neurodegeneration) to defects in the gastrointestinal innervation, such as Hirschsprung’s disease and other types of enteric dysganglionosis. Their common characteristics are a loss or lack of neuronal structures and functionality (Smith et al., 1984; Hagl et al., 2013) that cannot be overcome by healing processes.

Congenital neuropathic motility disorders of the gut are the reason behind rare and typically severe diseases in pediatric patients. These diseases vary from a local hypo- or aganglionosis of the terminal colon to infestation of the total gut. Whereas the typical disease in this field, Hirschsprung’s disease, affects one in 5000 live births (Amiel and Lyonnet, 2001), a complete aganglionosis of the gut, known as Zuelzer-Wilson-Syndrome, is found only in rare cases. Children suffering from this total absence of innervation with almost complete lack of gastrointestinal motility exhibit insufficient intestinal resorption capacitiy and have to rely on a lifelong complete parenteral nutrition. Despite significant progress in research, curative clinical therapies are not in sight and therapeutic options based on neural stem cell transplantation to restore neural and glial cells are still experimental. A common treatment in children suffering from partial aganglionosis is the resection of the affected bowel segments. As a new therapeutic option, transplantation of neuronal stem cells to aganglionotic gut segments seems to be a both promising and realistic option.

The enteric nervous system (ENS) harbors a potent and easily accessible autologous source of enteric neural stem cells (Young and Newgreen, 2001; Furness, 2006; Hagl et al., 2013) that are excellently suited for cell transplantation therapies (Hagl et al., 2013). Pre/postnatal and adult ENS contains a high number of neural stem cells all through its life span (Grundmann et al., 2019). These neural crest derived stem cells are able to respond and adapt to inner and outer influences throughout life time (Schäfer et al., 2009; Hagl et al., 2013). With these findings in mind, autologous cell therapy for neurological disorders of the gut and even the CNS should be further promoted, and enteric neural stem cells might be the perfect candidate for neural cell transplantation (Rauch et al., 2006; Hagl et al., 2013). In this context, a current issue to address is the fact that the number of cells needed to colonize the diseased area can be enormous, particularly in case of long-segment hypo- or aganglionosis. In this case especially, it might not be possible to harvest a sufficient number of neural stem cells in one procedure. Thus, repeated cycles of biopsies and neural stem cell expansion will be necessary to obtain a sufficient quantity of cells (Metzger et al., 2009). Although cryopreservation has been shown to be an option to store cells for longer periods of time, unfortunately it often has a severe impact on cell vitality (Pegg, 2007). Until now, there are various protocols and methods for freezing and thawing cells, each with inherent problems and benefits. Post-thaw viability tends to decrease with the complexity of tissues (Bojic et al., 2021) and survival rates after thawing depend on the method of freezing and vary for cells of different origin and species. Strategies for freezing have been modified over the last years to significantly improve cell survival. Post-thawing survival of human embryonic and fetal neural progenitor cells is described as 20%–40% (Ha et al., 2005), whereas survial can be increased up to 80% for stem cells of different origin (Nishiyama et al., 2016).

In general, cryopreservation strategies usually require a slow and continuous decrease of temperature during the freezing process to avoid structural damage caused by the formation of ice crystals throughout physical changes (Sambu, 2015). The two primary cryopreservation techniques are either well-established combinations of controlled rates of slow freezing or a newer process known as vitrification*,* where flash-freezing is applied.

In this context slow programmable freezing describes the gradual freezing with a controlled stepwise reduction of temperature (Vutyavanich et al., 2010). Here, lethal intracellular freezing can be avoided as long as cooling is slow enough to allow water to depart from the cell into the extracellular fluid. In addition, specific compounds can be used to impede intracellular ice crystal growth (Deller et al., 2014; Sambu, 2015). While a typical cooling rate of 1 °C/min is regarded acceptable for several mammalian cells when treated with cryoprotectants, such as dimethyl sulphoxide (DMSO) or glycerol, this is not an overall optimum and it differs between cells of various size and water permeability (Leibo and Mazur, 1971; Whittingham et al., 1972; Mazur et al., 2008; Baert et al., 2012).

In comparison, the process of vitrification (flash-freezing in the range of mega kelvins per second) describes a separate physical phenomenon that occurs within a small range of temperature described as the “glass transition temperature” around −80 to −130°C (Rall and Fahy, 1985; Kuleshova et al., 1999; Kuleshova et al., 2004; Bhat et al., 2005).

In vitrification, samples change from liquid to solid state without ice crystal formation (Bhat et al., 2005). For the clinical use of cryopreservation where sufficiently high cooling rates cannot be achieved due to sample size, flash-freezing typically requires the addition of cryoprotectants. However, the procedure of flash-freezing is more complicated than slow freezing and high concentrations of cryoprotectants may have a toxic impact on tissue. Additionally, only small cell volumes can be cryopreserved using this technique.

As an alternative to solutions containing DMSO, the cryopreservative agent StemCell Keep™ has been developed by Matsumura et al. (Matsumura and Hyon, 2009; Matsumura et al., 2010; Matsumura et al., 2011). The addition of polymeric additives inhibits devitrification and stabilizes the glassy state, a method that presents with low-toxicity (Mukaida et al., 1998) and offers the possibility to flash-freeze even larger amounts of stem cells. As a chemically defined and commercially available cryopreservation solution StemCell Keep™ has already proven effective for the cryopreservation of human-induced pluripotent stem cells (Matsumura et al., 2010).

In this study we aimed to find a method that minimizes harmful freezing effects with minimal influence on cell properties and at the same time allows cryopreservation of a large number of cells for future transplantation. Hence, we compared slow and rapid cryopreservation methods with different cryopreservation media for the storage of ENS-derived neurospheres (ENSdN). Furthermore, we investigated the impact of different freezing and thawing protocols upon enteric neural stem cell survival, protein and gene expression. Finally, using calcium microfluorimetry we demonstrated that cell functionality remained unaffected by the freezing procedure.

## Materials and methods

### Animals

Pregnant Sprague Dawley rats were obtained from JANVIER LABS and newborn pups used for experiments. This study was carried out in strict accordance with the recommendations for the care and use of laboratory animals of the German animal protection law. Animal experiments were approved by the internal Veterinary Inspection Office in Mannheim. Authorization number: I-29/23 R.

### Generation of ENS-derived neurospheres

Newborn rat pups (age 4–6 days) were decapitated, dissected and the whole intestine removed and immediately stored on ice in MEM (Gibco, Thermo Fischer Scientific) containing antibiotics as previously described (Schäfer and Mestres, 1999; Schäfer et al., 2003; Grundmann et al., 2015). Then stripped muscle layers were incubated in a collagenase II solution (Worthington Biochemical Corporation) for 2 h. After vortexing the tissue for 20 s, the suspension was filtered through a sterile syringe filter (cell strainer, BD biosciences), pore size 40 μm. The supernatant was centrifuged three times. The obtained myenteric plexus was incubated in accutase (PAA Laboratories) for 20 min and dissociated by trituration. In a second centrifugation step, the accutase was removed and replaced with 10 mL culture medium adapted for expansion (Neurobasal A (Gibco), supplemented with 2% B27- (without vitamin A, Gibco), EGF (10 ng/mL, Tebue), bFGF (20 ng/mL; Tebue) and GDNF (10 ng/mL, Tebue), 1% albumin (Sigma-Aldrich), 0.25% 2-mercaptoethanol (50 mM, Invitrogen), 0.12% glutamine (200 mM, Sigma-Aldrich), gentamycin/metronidazole (100 μg/mL) or 1% penicillin/streptomycin (PAA Laboratories)). Cells were kept at standardized densities (1 × 10^6^ cells/25 cm^2^ flask). After 2 days of culture *in vitro*, the first floating neurospheres were seen, while neurons and radiating glial cells expanded at the bottom (Hagl et al., 2013). Cells were harvested after 3 days of culture after formation of small and medium sized neurospheres.

### Cryopreservation conditions and protocols

**FIGURE 1:**
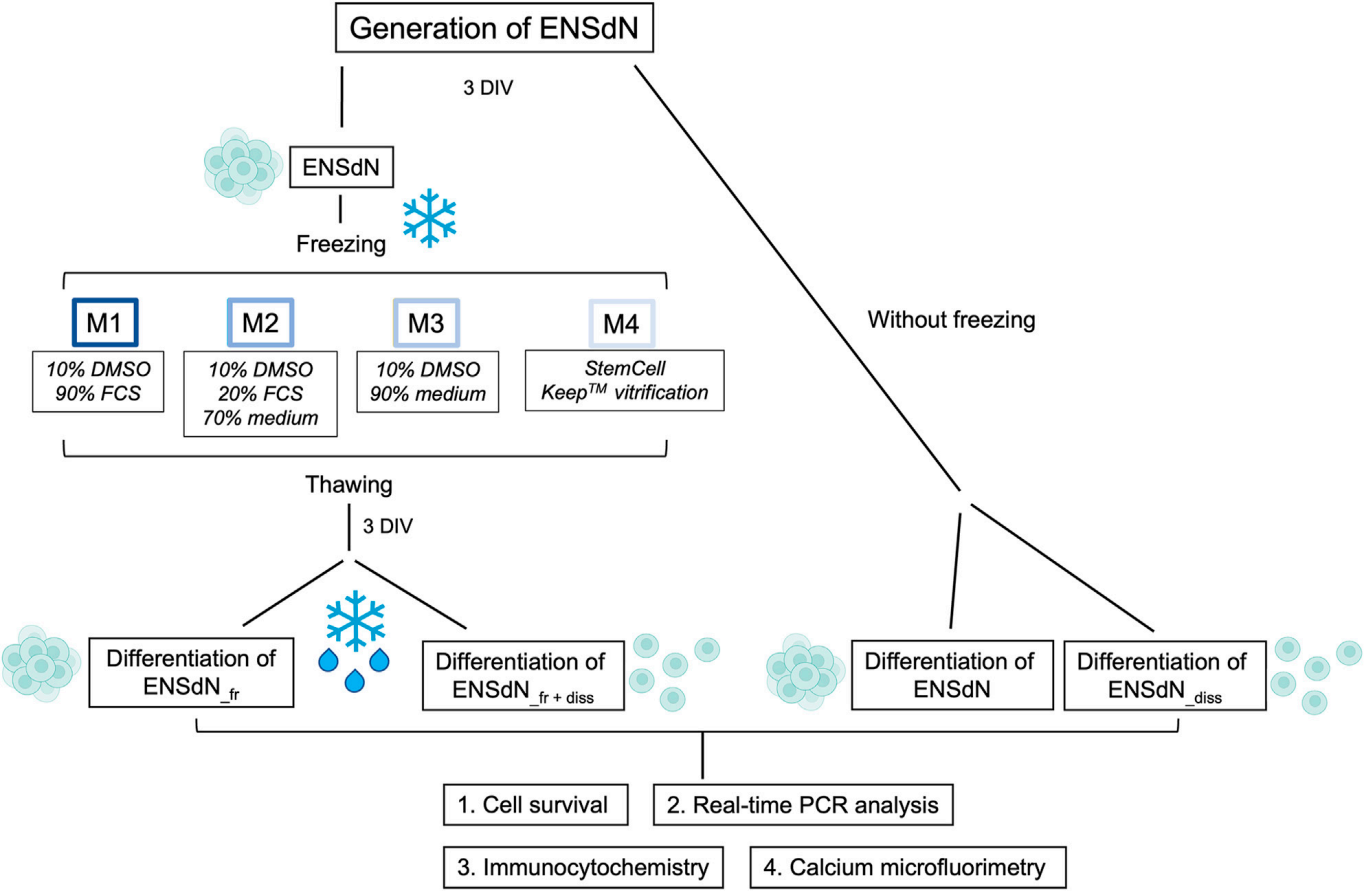
Study flow chart: Individual cryopreservation conditions and methods for the investigation of ENSdN’s survival, gene and protein expression and cell function (DIV = days *in vitro*).

ENSdN were either frozen at a controlled rate of slow freezing or based on a flash-freezing process. We used four different media with/without DMSO and maintained ENSdN for 2 weeks at −180°C (Figure 1).

In preparation for freezing ENSdN were centrifuged, the supernatant removed and the cell pellet was resuspended in the corresponding freezing medium.

• Condition 1: M1 = 90% Fetal Calf Serum (FCS) + 10% DMSO.
• Condition 2: M2 = 20% FCS +70% culture medium (Neurobasal A (Gibco), supplemented with 2% B27- (without vitamin A, Gibco), 1% albumin (Sigma-Aldrich), 0.25% 2-mercaptoethanol (50 mM, Invitrogen), 0.12% glutamine (200 mM, Sigma-Aldrich), 1% penicillin/streptomycin (PAA Laboratories)) + 10% DMSO.
• Condition 3: M3 = 90% culture medium +10% DMSO.
• Condition 4: M4 = StemCell Keep™ (BioVerde) without DMSO, serum or protein.

For conditions 1–3 we used approximately 1.5 × 10^6^ cells in DMSO containing cryopreservation medium and the slow-freezing method with a cooling rate of 1 °C/min in a freezing container.

Condition 4 combines a DMSO and FCS-free cryopreservation medium (StemCell Keep™, BioVerde) with the process of flash-freezing (vitrification)*.* We used 1.5 × 10^6^ cells in five cryovials, containing 200 µL cryopreservation medium (M4) each, that were immediately frozen by superfast cooling in liquid nitrogen. Cells were stored in liquid nitrogen for 2 weeks before using them for *in vitro* experiments.

### Thawing process

Condition 1–3:

ENSdN were thawed in the cryovial in a water bath at 37 °C and transferred in culture medium.

Condition 4:

For thawing of cells treated under condition M4, 1 mL of culture medium (37 °C) was added to each cryovial, resuspended and then transferred to 4 mL culture medium.

### Differentiation of ENS-derived neurospheres

After 3 days of proliferative culture, the floating ENSdN (fresh or thawed) were harvested and utilized either for differentiation directly or dissociated in accutase (PAA Laboratories GmbH) for 20 min and triturated using consecutively 23 and 27 gauge needles, 3 times each. To avoid shear stress, aspiration of the tissues was performed slowly. After dissociation, the cells were centrifuged (120x g, 5 min), the supernatant removed and the pellet resuspended in 1 mL medium. The intact or dissociated ENSdN were plated on poly-L-lysine-laminin coated coverslips in 24 or 12 well plates (Becton Dickinson) in culture medium for differentiation (Neurobasal A (Gibco) supplemented with 2% B27+ (with vitamin A, Gibco), 1% albumin (Sigma-Aldrich), 0.25% 2-mercaptoethanol (50 mM, Invitrogen), 0.12% glutamine (200 mM, Sigma-Aldrich), GDNF (10 ng/mL, Tebue), neuturin (10 ng/mL Tebue), gentamycin and metronidazole (100 μg/mL) and 1% penicillin/streptomycin (PAA Laboratories GmbH)). In case of functional imaging, 10% of FCS (Gibco) was added to the medium in order to help cell attachment.

### Cell survival assay with propidium iodide

For cell survival analysis, the cells were stained with propidium iodide (PI, 5 μg/mL) for 10 min at 37°C with 5% CO_2_. Then the staining solution was removed and the samples were washed two times with PBS. Samples were fixed with 4% formaldehyde for 10 min. The cultures were washed twice with PBS and then incubated with DAPI (Sigma, 0.3 μg/mL), which was used as nuclear stain.

To quantify the cells, PI stained (dead cells) cell numbers were assessed and related to the total cell number, based on nuclear DAPI stains (total cell number).

### Real-time PCR analysis of gene expression

Total cellular RNA from freshly isolated myenteric plexus was extracted directly (d0) or after cultivation in proliferation medium for 3 days (d3) using an RNA Micro Isolation Kit (Bioline) following the manufacturer’s protocol. RNA concentration was determined spectrophotometrically with the infinite M200 micro plate reader (Tecan) and RNA quality assessment was tested with RNA 6000 Nano Kit (Agilent Technologies) and the Agilent Bioanalyzer 2100 (Agilent Technologies). BioScript TM was used to generate cDNAs (Bioline). For real time PCR the SensiMixSYBR Low-Rox Kit (Bioline) was used on a MX3005 (Stratagene). Glyceraldehyde-3-phosphate dehydrogenase (GAPDH) and 18 sRNA primers were used as an internal standard. The PCR conditions were as follows: initial denaturation 10 min, 95°C, 40 cycles of denaturation, 30 s, 95°C; annealing 30 s, 58°C; 30 s, 72°C. Primer sequences were used as listed below (Table 1). Data were normalized for GAPDH mRNA expression using the delta-delta-CT method (Livak and Schmittgen, 2001).

**TABLE 1:** Primer sequences for real-time PCR.

Primer		Sequence		Design
hmr-r18SrRNA-fw	5'-	CTT​TGG​TCG​CTC​GCT​CCT​C	−3′	
hmr-r18SrRNA-rv	5'-	CTG​ACC​GGG​TTG​GTT​TTG​AT	−3′	
r-S100B fw	5'-	TTG​CCC​TCA​TTG​ATG​TCT​TCC​A	−3′	Pavel D. Lisachev, 2010
r-S100B rv	5'-	TCT​GCC​TTG​ATT​CTT​ACA​GGT​GAC	−3′	Pavel D. Lisachev, 2010
r-GAPDH fw	5'-	GTA​TGA​CTC​TAC​CCA​CGG​CAA​GT	−3′	F. DU, 2009
r-GAPDH rv	5'-	TTC​CCG​TTG​ATG​ACC​AGC​TT	−3′	F. DU, 2009
r-PGP9.5 fw	5'-	CCC​TGA​AGA​CAG​AGC​CAA​GTG	−3′	F. DU, 2009
r-PGP9.5 rv	5'-	GAG​TCA​TGG​GCT​GCC​TGA​A	−3′	F. DU, 2009
r-GFAP fw	5'-	ACC​TCG​GCA​CCC​TGA​GGC​AG	−3′	Nobuki Matsuura 2001
r-GFAP rv	5'-	CCA​GCG​ACT​CAA​CCT​TCC​TC	−3′	Nobuki Matsuura 2001
r-tubulin b-III fw	5'-	AGA​CCT​ACT​GCA​TCG​ACA​ATG​AAG	−3′	Telma T. 2010
r-tubulin b-III rv	5'-	GCT​CAT​GGT​AGC​AGA​CAC​AAG​G	−3′	Telma T. 2010
r-Nestin fw	5'-	GGA​GTG​TCG​CTT​AGA​GGT​GC	−3′	Lendahl et al. (1990)
r-Nestin rv	5'-	CAG​CAG​AGT​CCT​GTA​TGT​AGC​C	−3′	Lendahl et al. (1990)
r-Jmjd4 fw	5'-	AGC​CTG​GCG​AGA​TGG​TGT​TTG	−3′	SP Yenamandra, SD Darekar et al. (2012)
r-Jmjd4 rv	5'-	GCC​ATT​GAC​CCA​GTT​GTG​GT	−3′	SP Yenamandra, SD Darekar et al. (2012)
r-Nanog fw	5'-	TTG​GAA​CGC​TGC​TCC​GCT​CC	−3′	SP Yenamandra, SD Darekar et al. (2012)
r-Nanog rv	5'-	CGC​CTG​GCT​TTC​CCT​AGT​GGC	−3′	SP Yenamandra, SD Darekar et al. (2012)
r-Oct4 fw	5'-	GGA​GGG​ATG​GCA​TAC​TGT​GGA​CCT	−3′	SP Yenamandra, SD Darekar et al. (2012)
r-Oct4 rv	5'-	TCC​TGG​GAC​TCC​TCG​GGA​CTA​GG	−3′	SP Yenamandra, SD Darekar et al. (2012)
r-Sox2 fw	5'-	ACT​AAT​CAC​AAC​AAT​CGC​GGC​GGC	−3′	SP Yenamandra, SD Darekar et al. (2012)
r-Sox2 rv	5'-	GAC​GGG​CGA​AGT​GCA​ATT​GGG​A	−3′	SP Yenamandra, SD Darekar et al. (2012)
r-p75- fw	5'-	ACC​ACC​GAC​AAC​CTC​ATT​CC	−3′	Bing He, et al. (2012)
r-p75- rv	5'-	CAC​TGT​CGC​TGT​GCA​GTT​TC	−3′	Bing He, et al. (2012)

### Immunocytochemistry

Differentiated cultures were fixed with formaldehyde, followed by 45 min permeabilization in 0.5% triton X-100 and then blocked in 10% normal goat serum (DAKO) for 1 h. The corresponding primary antibodies for Nestin (Millipore, mouse-anti-Nestin [1:200]), Nanog (R&D System, goat-anti-Nanog [1:20]), Sox2 (R&D System, mouse -anti-Sox2 [1:50]), Oct4 (Abcam, rabbit-anti-Oct4 [1:200]), ß-Tubulin III (Millipore, mouse-anti-Tubulin [1:1000]), GFAP (Dako, rabbit-anti-GFAP [1:100]), S100 (Dako, rabbit-anti-Rabbit-S100 [1:250]), PGP9.5 (Dako, rabbit-anti-PGP9.5 [1:250]) and p75 (Abcam, mouse-anti-p75 [1:500]) were added and incubated over night at 4 °C. Samples were washed three times with PBS and incubated with the corresponding secondary antibodies (Alexa^®^ 488 anti-mouse-IgG, Alexa^®^ 488 anti-rabbit-IgG, Alexa^®^ 488 anti-goat-IgG for 4 h at room temperature (RT). 4′, 6-diamidino-2-phenylindole (DAPI) (Sigma-Aldrich, 0.3 μg/mL) was used as nuclear stain. Cell numbers were related to an overall cell count based on DAPI stainings.

### Microscopy

Intact and dissociated ENSdN on coverslips were visualized using a BIOREVO BZ-9000 microscope (Keyence) and the analysis software BZ-II Analyzer (Keyence). Image processing was performed with the open source program Gimp.

### Calcium microfluorimetry of ENS-derived neurospheres

Single-cell intracellular Ca^2+^ measurements of ENSdN were performed after the differentiation of unfrozen or frozen neurospheres treated with condition M1 (ENSdN__frM1_). For differentiation, cells were plated on PLL (Poly-L-Lysine, Sigma-Aldrich; 1 mg/mL) and ECM (ECM Gel, Sigma-Aldrich; dilution 1:100 in DMEM) coated coverslips for better adhesion in 12-well plates until density was about 1/3 of the coverslip. For measurements of [Ca^2+^]_i_, cells were loaded with 3 µL FURA-2AM (Calbiochem) and the same amount (in µl) of Pluronic F-12775 (Calbiochem) for 45–60 min in Tyrode’s solution (148 mM NaCl, 5 mM KCl, 1 mM MgCl2, 2 mM CaCl2, 10 mM HEPES; 10 mM glucose; adjusted to pH 7.38 with NaOH). After washing with Tyrode’s solution for 30 min, cells were mounted on the stage of an inverted microscope (Olympus IX81 equipped with cellR Imagingsystem; Olympus) in an open bath chamber (Series 40 Chamber; Warner Instruments) and superfused by Tyrode’s solution (1–3 mL/min) at room temperature (22°C–24 °C). Fluorescent signals of the cells were evoked by alternating illumination (340/380 nm wavelength) and respective fluorescent signals (510 nm) detected by an ORCA-R2 camera (Hamamatsu Photonics). The ratio of emission for 340/380 nm excitation was used as relative change in [Ca^2+^]_i_. For analysis, cells were marked as regions of interest (ROIs) and changes in [Ca^2+^]_i_ during the period of stimulus application were calculated as difference between maximum value and basal value of corresponding cells. Cells were assorted to subgroups according to responsiveness to different stimuli (nicotine 50 μM, acetylcholine 50 μM, ATP 100 µM (all Sigma Aldrich), K^+^ solution (140 mM)).

### Statistics

Results are demonstrated as mean ± standard error of the mean (SEM). Data were visually evaluated for normal distribution using quantile-quantile (Q-Q) plots and tested for statistical significance using unpaired Student’s t-test for unequal variances (two-tailed). *p*-values <0.05 were considered significant (* = *p* < 0.05, ** = *p* < 0.01, *** = *p* < 0.001).

## Results

### Comparison of cell survival of unfrozen and frozen enteric nervous system-derived neurospheres

**FIGURE 2:**
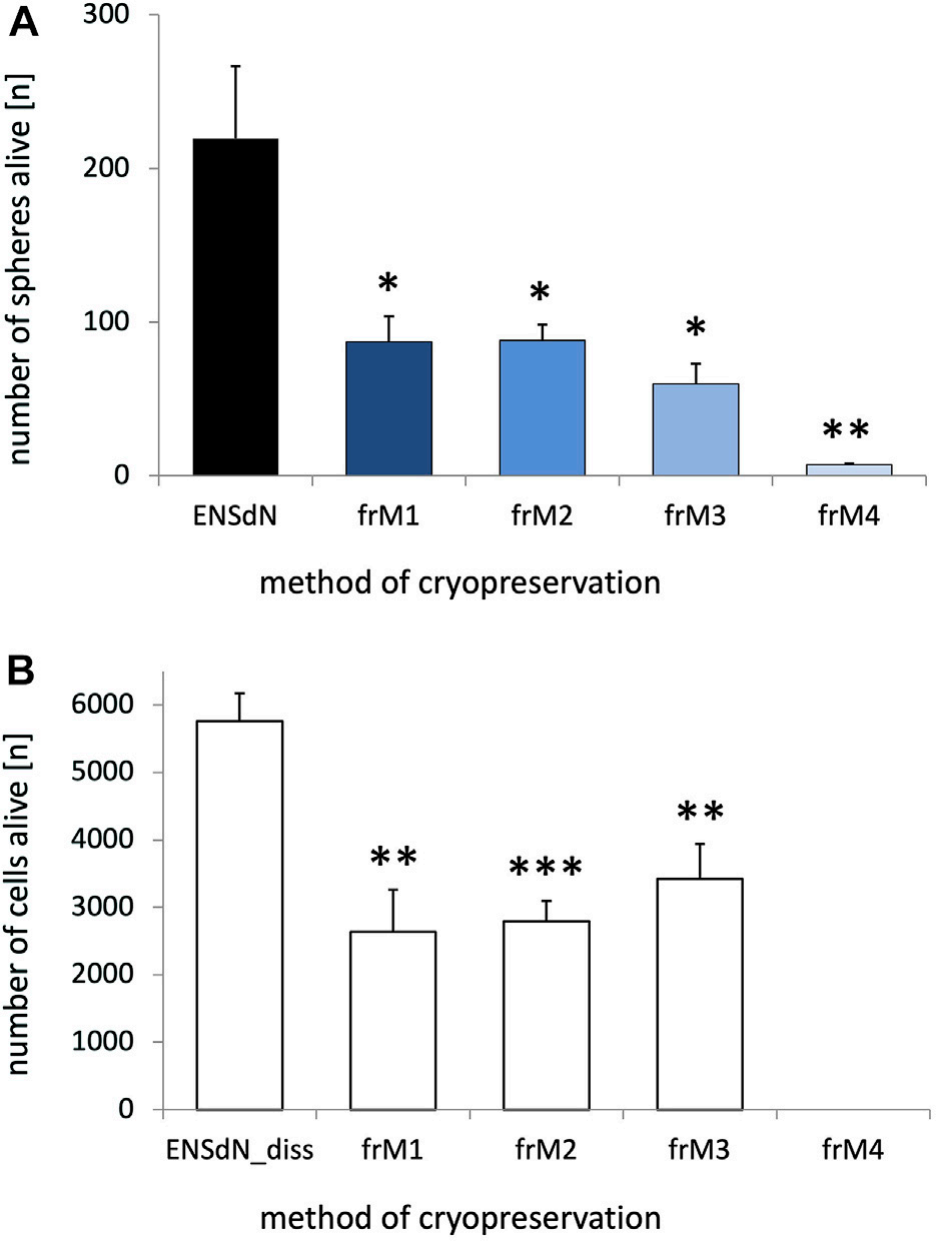
Survival of ENSdN and ENSdN__diss_ before and after freezing with different freezing methods and media. ENSdN were cultivated 3 days *in vitro* and then seeded onto 24 well-plates either directly as spheres (ENSdN, **(A)**) or dissociated (ENSdN__diss_, **(B)**); or were frozen with condition M1-M4 (ENSdN__fr_ and ENSdN__fr+diss_). Number of living spheres **(A)**/cells **(B)** was assessed by DAPI staining after 3 days of cultivation. Percentage of death spheres/cells was assessed by propidium iodide assay (n = 6 for dissociated cells and n = 3-8 for spheres; * = *p* < 0.05, ** = *p* < 0.01, *** = *p* < 0.001, Student’s t-test unfrozen vs frozen M1-4/3; data are shown as mean ± SEM).

The influence of freezing on the survival of enteric nervous system derived neurospheres (ENSdN) was assessed using a live-dead-assay, based on combined propidium iodide (PI) and DAPI staining. Number of ENSdN before (ENSdN, ENSdN_diss) and after freezing (ENSdN_fr, ENSdN_fr + diss) was analyzed (Figure 2).

Our results show that the process of freezing significantly reduced the number of surviving ENSdN. The average number of living intact ENSdN was 219.5 (±47.1) before freezing. Both M1 and M2 freezing protocols resulted in a similar reduction of spheres (ENSdN__fr_) up to 87.2 (±16.8) or 88.2 (±10.5) respectively. After freezing with M3 the outcome was even worse and delivered only 60.0 (±12.9) surviving spheres. M4 protocol nearly led to a complete loss of tissue with a number of only 7.3 (±0.7) surviving ENSdN__fr_ (Figure 2A, n = 8/6/6/6/3; *p* = 0.0273, *p* = 0.0271, *p* = 0.0113 and *p* = 0.0027 for ENSdN vs ENSdN__frM1, M2, M3, M4_; SEM).

When ENSdN were dissociated fresh or after freezing and cultured as single cells (ENSdN__diss_) the number of surviving cells after freezing with protocol M1-M4 was also reduced significantly. We counted a total number of 5760.5 (±416.3) living cells per coverslips for unfrozen ENSdN__diss_, compared to 2635.2 (±625.4), 2795.3 (±298.5) and 3425.0 (±516.3) cells for ENSdN__fr+dissM1, M2, and M3_, respectively (*p* = 0.0026, *p* = 0.0002 and *p* = 0.0059; n = 6). Cells frozen following the M4 procedure did not survive at all after dissociation (Figure 2B).

However, the percentage of surviving cells was slightly higher when cells were cultured subsequent to dissociation compared to cultures of intact spheres.

To summarize, slow freezing conditions M1-M3 resulted in higher rates of surviving cells compared to flash-freezing (M4) in both intact and dissociated neurospheres. Overall survival was slightly higher when cells were cultured dissociated.

### Evaluation of RNA expression of frozen and unfrozen enteric nervous system derived neurospheres

DMSO is known to induce differentiation in embryonic stem cells and initiates downregulation of stem cell markers such as Nanog, Nestin, Sox-2 and Oct-4 in a concentration-dependent manner (Adler et al., 2006; Pal et al., 2012). In order to investigate the effect of DMSO-containing freezing media on RNA expression in ENSdN, we subsequently analyzed RNA expression profiles of neuronal, glial and stem cell marker genes (PGP9.5, ß-TubulinIII, GFAP, S100, p75, Nestin, Nanog, Oct4 and Sox2).

**FIGURE 3:**
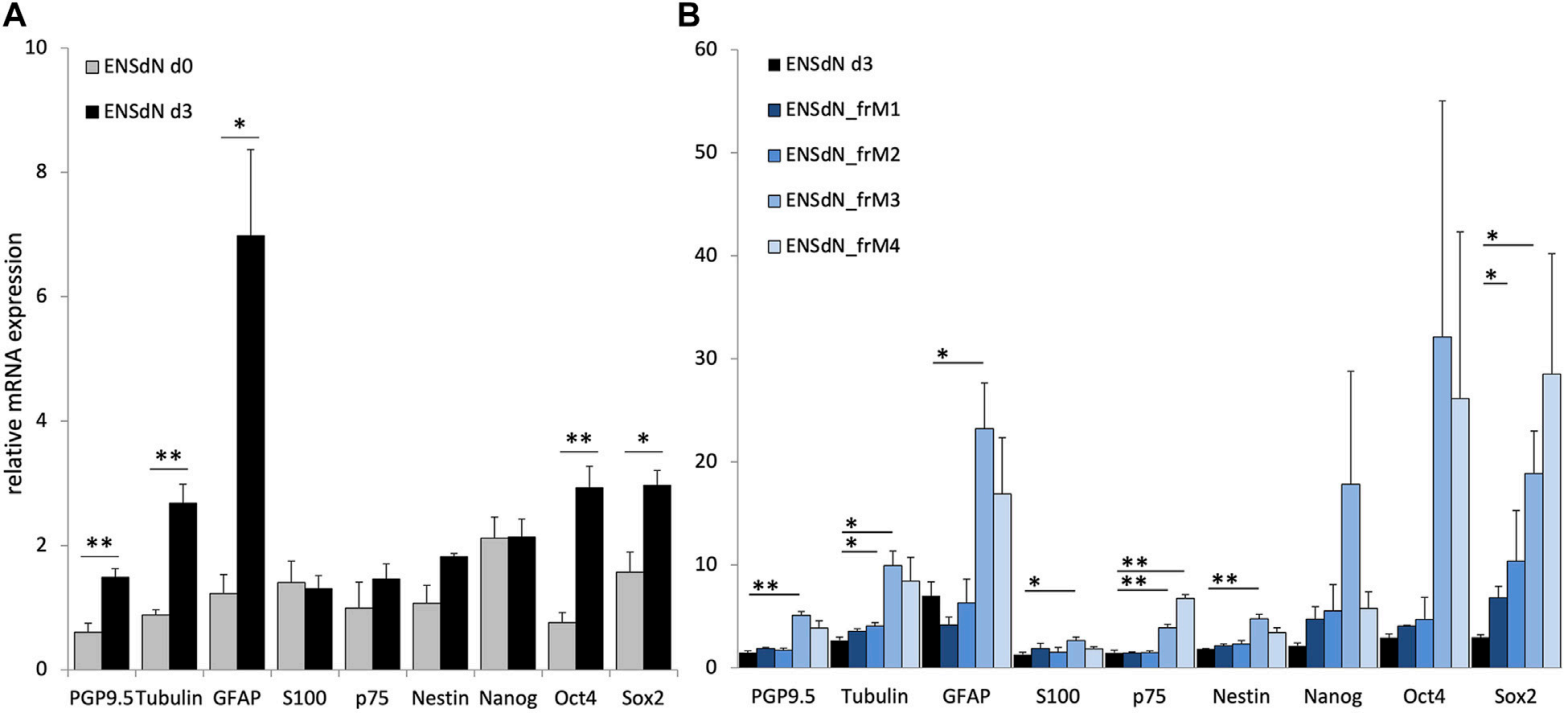
RNA expression of proliferating ENSdN and the influence of different freezing conditions on ENSdN. **(A)**. RNA pattern of isolated myenteric plexus cells (d0) were compared to ENSdN that have been cultivated for 3 days (d3). **(B)**. Relative mRNA expression of neural, glial and stem cell markers without and after freezing with different conditions (M1-M3). Data were normalized for GAPDH mRNA expression using the delta-delta-CT method (Adler et al., 2006). Data are shown as mean ± SEM and tested for statistical significance using Student’s t-test d0 vs d3 **(A)** and unfrozen d3 vs frozen M1-M4 **(B)** (n = 4–6; * = *p* < 0.05, ** = *p* < 0.01, *** = *p* < 0.001).

In a first step, change in RNA expression during the process of cell cultivation was investigated. RNA expression profile from freshly isolated myenteric plexus (d0) was compared to cells that were cultured in proliferation medium for 3 days (d3; Figure 3A).

We could observe that growing cells in proliferation culture promoted relative mRNA expression of PGP9.5 and ß-TubulinIII from 0.60 (±0.14) to 1.48 (±0.14) and from 0.88 (±0.08) to 2.68 (±0.31), respectively (*p* = 0.0027 and *p* = 0.0049; SEM). GFAP expression increased from 1.23 (±0.31) to 6.98 (±1.39) and the expression of the two stem cell marker Oct4 and Sox2 was increased from 0.76 (±0.16) to 2.93 (±0.34) and 1.57 (±0.32) to 2.97 (±0.24) (*p* = 0.0406, 0.0088, 0.0369, respectively).

In order to investigate the additional effect of different freezing methods on mRNA expression of ENSdN, mRNA expression profiles of frozen ENSdN (ENSdN__fr_) were compared to the expression profile of intact ENSdN after 3 days of proliferation culture. For ENSdN__frM1 and M2_ we observed a significant elevation of Sox2 mRNA levels for M1 (*p* = 0.0237) and ß-TubulinIII for M2 (*p* = 0.0160, Figure 3B). For ENSdN__frM3_ expression of almost all genes was significantly increased. The expression of neural markers PGP9.5 and ß-Tubulin III was increased from 1.48 (±0.14) to 5.08 (±0.38) and from 2.68 (±0.31) to 9.92 (±1.43); *p* = 0.0017 and 0.0177. Glial marker expression was also promoted from 6.98 (±1.39) to 23.22 (±4.44) for GFAP and from 1.30 (±0.22) to 2.63 (±0.35) for S100; *p* = 0.0404 and 0.0325. Expression of stem cell markers was significantly increased from 1.46 (±0.24) to 3.88 (±0.35) for p75, 1.82 (±0.05) to 4.76 (±0.45) for Nestin and 2.97 (±0.24) to 18.86 (±4.11) for Sox2, *p* = 0.0026, 0.0093 and 0.0403. When the M4 freezing condition was used, similar mRNA expression profiles as for condition M3 could be observed with a significant increase in stem cell marker p75 (*p* = 0.0012). However, the glial and neuronal marker expression missed the level of significance.

In conclusion, RNA expression profiles of ENSdN showed that the expression of neural, glial and stem cell markers changes during the process of proliferation. Freezing of ENSdN using condition M1 and M2 had less effect on RNA expression profiles compared to condition M3/4.

### Assessment of protein expression of differentiated frozen and unfrozen enteric nervous system derived neurospheres

**FIGURE 4:**
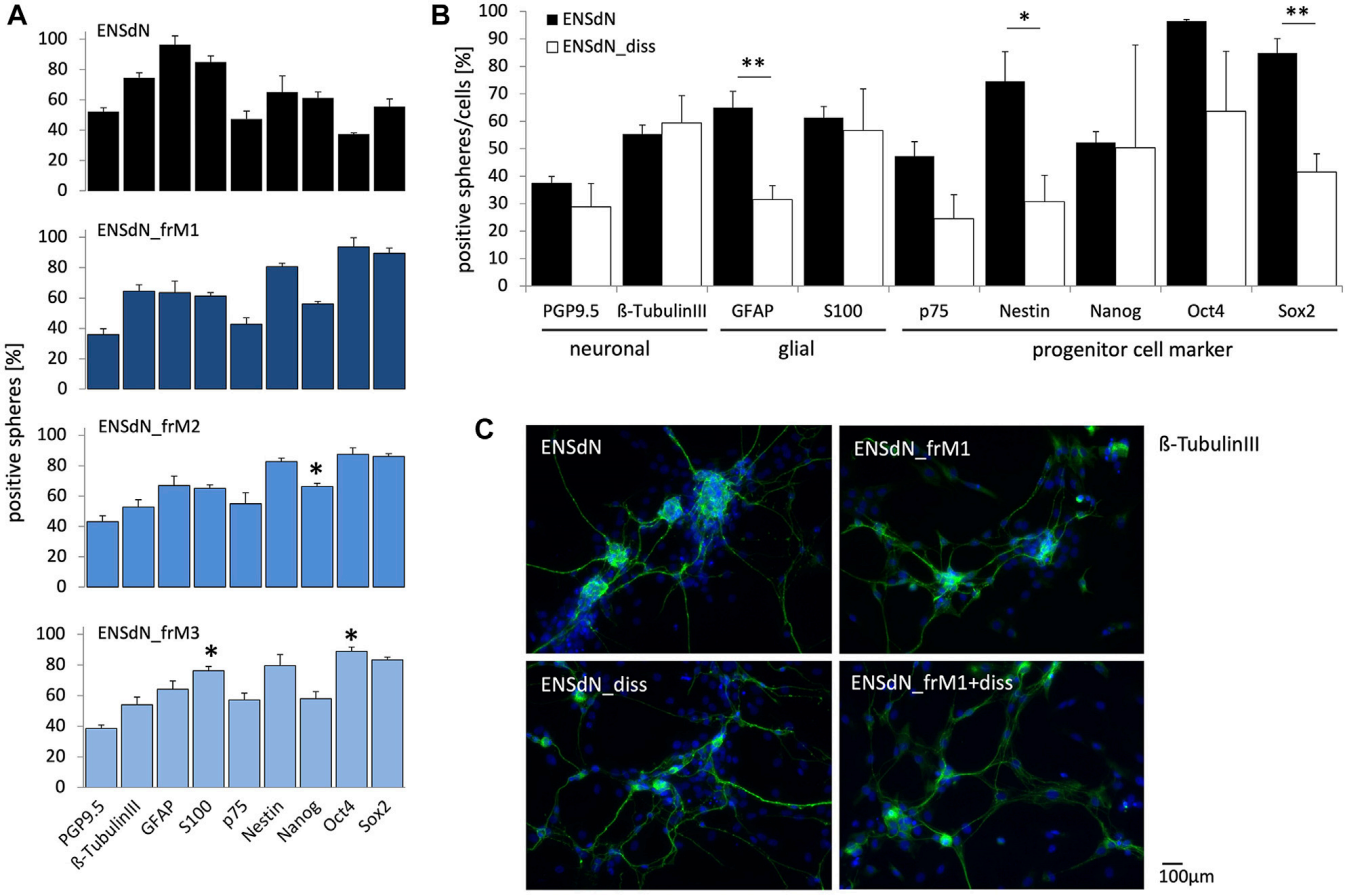
Protein expression of intact ENSdN and dissociated ENSdN without and after freezing with different freezing conditions. **(A)**. Quantification of protein expression of PGP9.5, β-TubulinIII, GFAP, S100, p75, Nestin, Nanog, Oct4 and Sox2 in ENSdN and ENSdN__frM1, M2 and M3_ (n = 4-6, Student’s t-test unfrozen vs frozen M1-M3). **(B)**. Neuronal, glial and progenitor cell protein expression in unfrozen ENSdN vs ENSdN__diss_ (n = 4-6, Student’s t-test ENSdN vs ENSdN__diss_, * = *p* < 0.05, ** = *p* < 0.01, *** = *p* < 0.001; data are shown as mean ± SEM). **(C)**. Protein expression patterns of neuronal marker ß-TubulinIII in ENSdN, ENSdN__frM1,_ ENSdN__diss_ and ENSdN__frM1+diss_.

To evaluate the effect of freezing on differentiated cultures of ENSdN, the expression of different neuronal, glial, and progenitor markers (PGP9.5, ß-TubulinIII, GFAP, S100, p75, Nestin, Nanog, Oct4 and Sox2) was assessed using immunohistochemical stainings. ENSdN were cultured and differentiated either as intact neurospheres (ENSdN) or after dissociation as single cells (ENSdN__diss_) and compared to frozen neurospheres (ENSdN__fr_) or cells (ENSdN__fr+diss_) (Figure 4).

For ENSdN we could observe the following protein distribution: 37.48% (±2.42%) of the cells were PGP9.5-positive, 55.43% (±3.20%) displayed a ß-TubulinIII staining, 65.01% (±5.85%) were GFAP-positive and 61.35% (±4.06%) expressed the S100 protein. Concerning neural stem cell markers we found 47.32% (±5.22%) of the cells p75-positive, 74.56% (±10.82%) displayed expression of nestin, while 52.29% (±3.92%), 96.41% (±0.67%) and 84.84% (±5.22%) presented Nanog, Oct4 and Sox2, respectively. Freezing of ENSdN with condition M1 (ENSdN__frM1_) did not alter protein expression at all, while M2 protocol led to a significant upregulation of Nanog (66.29% (±2.03%), *p* = 0.0142). ENSdN__frM3_ displayed a significant upregulation of Oct4 (88.84% (±2.76%), *p* = 0.0398), combined with a significant upregulation of S100 (76.13% (±2.94%), *p* = 0.0194; Figure 4A). Due to the lack of a considerable number of surviving ENSdN__frM4_, no protein profiles could be assessed for this condition.

To investigate the impact of dissociation of neurospheres prior to differentiation, we likewise investigated the protein patterns of dissociated ENSdN (ENSdN__diss_).

When we compared protein expression of ENSdN and ENSdN__diss_ without freezing, dissociated cells displayed a significant lower expression of GFAP, Nestin and Sox2 (Figure 4B, *p* = 0.0015, 0.0191 and 0.0025, respectively).

In contrast to protein expression patterns in intact ENSdN cultures, there were no significant differences between frozen (ENSdN__fr+dissM1, M2, M3_) and unfrozen cultures of dissociated cells (ENSdN__diss_), except for ENSdN__fr+dissM1_, where Sox2 was significantly upregulated to (71.59% (±8.44%), *p* = 0.0231, data not shown).

Comparing the different freezing methods, only freezing condition M1 did not alter the protein expression patterns of ENSdN.

The process of dissociation of ENSdN altered the protein expression, which was not further affected by additional freezing.

### Functional characterization of frozen and unfrozen enteric nervous system derived neurospheres

To investigate the effect of freezing and thawing on cell function, we compared intracellular calcium responses using calcium imaging. Since freezing condition M1 showed good survival rates and did not significantly alter gene as well as protein expression patterns in differentiated ENSdN in our setting, we chose ENSdN__frM1_ for functional imaging. Adherent cells derived from ENSdN without freezing or after freezing and thawing under condition M1 (ENSdN__frM1_) were plated, cultivated and differentiated on glass coverslips for 3 days, until they formed a layer and networks of neurons and glia in ganglion-like structures that were visible under differential interference contrast (DIC) microscopy.

**FIGURE 5:**
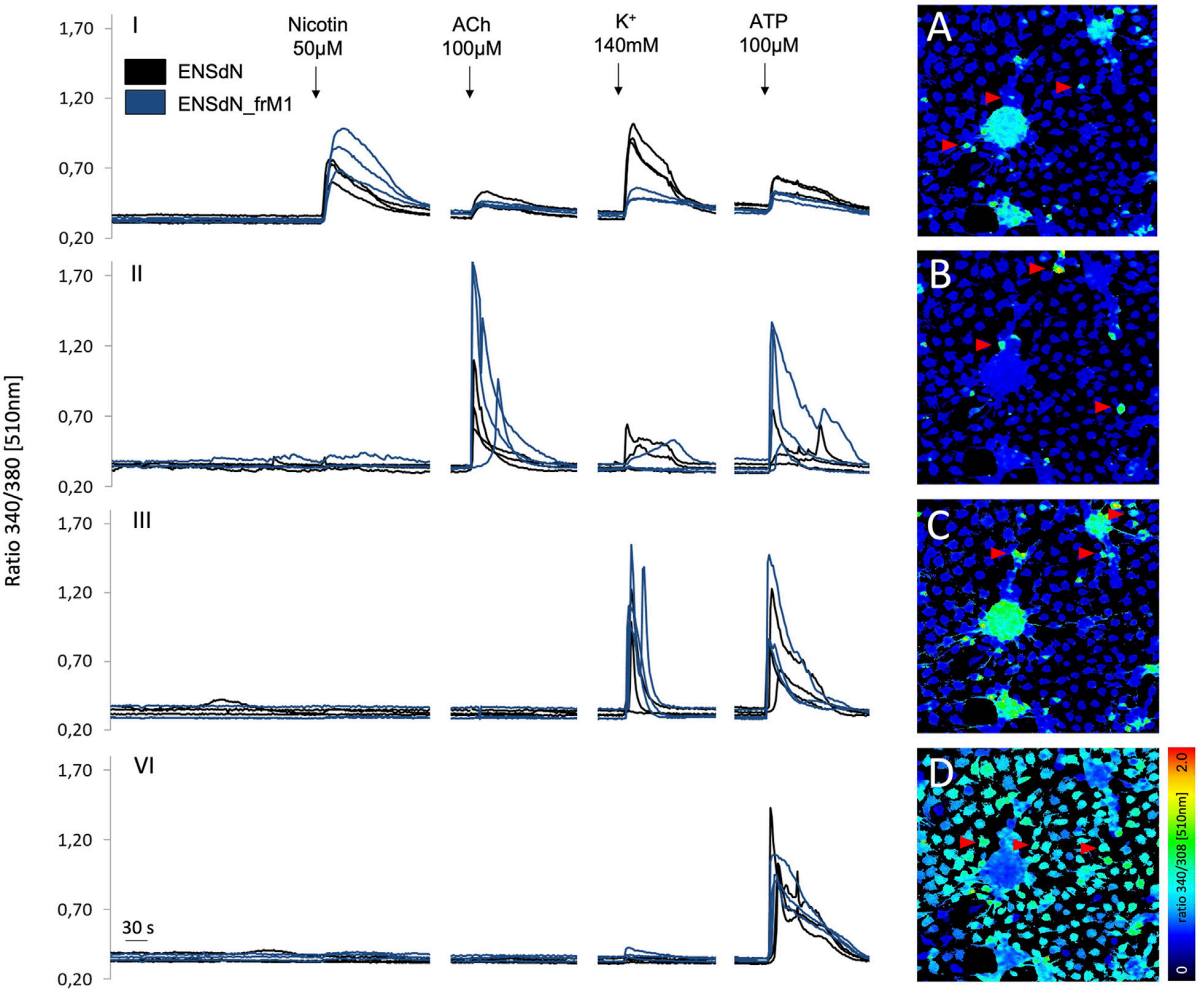
Changes in fluorescence ratio of ENSdN and ENSdN__frM1_ reveal different functional subgroups. Ratio 340/380 nm [510 nm] of representative cells (ENSdN (black lines, red arrows) and ENSdN__frM1_ (blue lines)) after stimulation with nicotine 50 μM, acetylcholine (ACh) 100 μM, K^+^ 140 mM and ATP 100 µM (black arrows). Group I-IV: cells were subdivided according to response patterns; **(A–D)**: pseudocoloured images of ENSdN I-IV, representing first increase in [Ca^2+^]_i_ after stimulation; A = nicotine, B = acetylcholine, C=K^+^, D = ATP.

Cells on coverslips cultivated as ENSdN or ENSdN__frM1_ were tested for differential calcium responses to nicotine (50 µM), acetylcholine (50 µM), ATP (100 µM) and high K^+^ (140 mM) for 60 s sequentially (n = 11 coverslips; 56.5 (±4.9) ROIs/coverslip for ENSdN vs n = 8 coverslips; 57.6 (±5.6) ROIs/coverslip for ENSdN__frM1_). However, subsequent application of nicotine, acetylcholine, K^+^ and ATP revealed different response patterns of cells. According to the response pattern of each single cell, cells were assorted to four different groups (Table 2; Figure 5):

**TABLE 2:** Group assortment according to cell response patterns to different neurochemical stimuli.

	Nicotine	Acetylcholine	K^+^	ATP
Group I	+	+	+	+
Group II	-	+	+	+
Group III	-	-	+	+
Group IV	-	-	-	+

Group I: Cells responding to nicotine also displayed a rise in [Ca^2+^]_i_ when challenged with acetylcholine, high K^+^ and ATP subsequently. Group II:. Cells responding to acetylcholine (without prior response to nicotine) also displayed a rise in [Ca^2+^]_i_ when challenged with high K^+^ and ATP subsequently. Group III: Cells responding to high K^+^ (without prior response to nicotine and acetylcholine) also displayed a rise in [Ca^2+^]_i_ when challenged with ATP subsequently. Group IV: Cells responding to ATP only.

**FIGURE 6:**
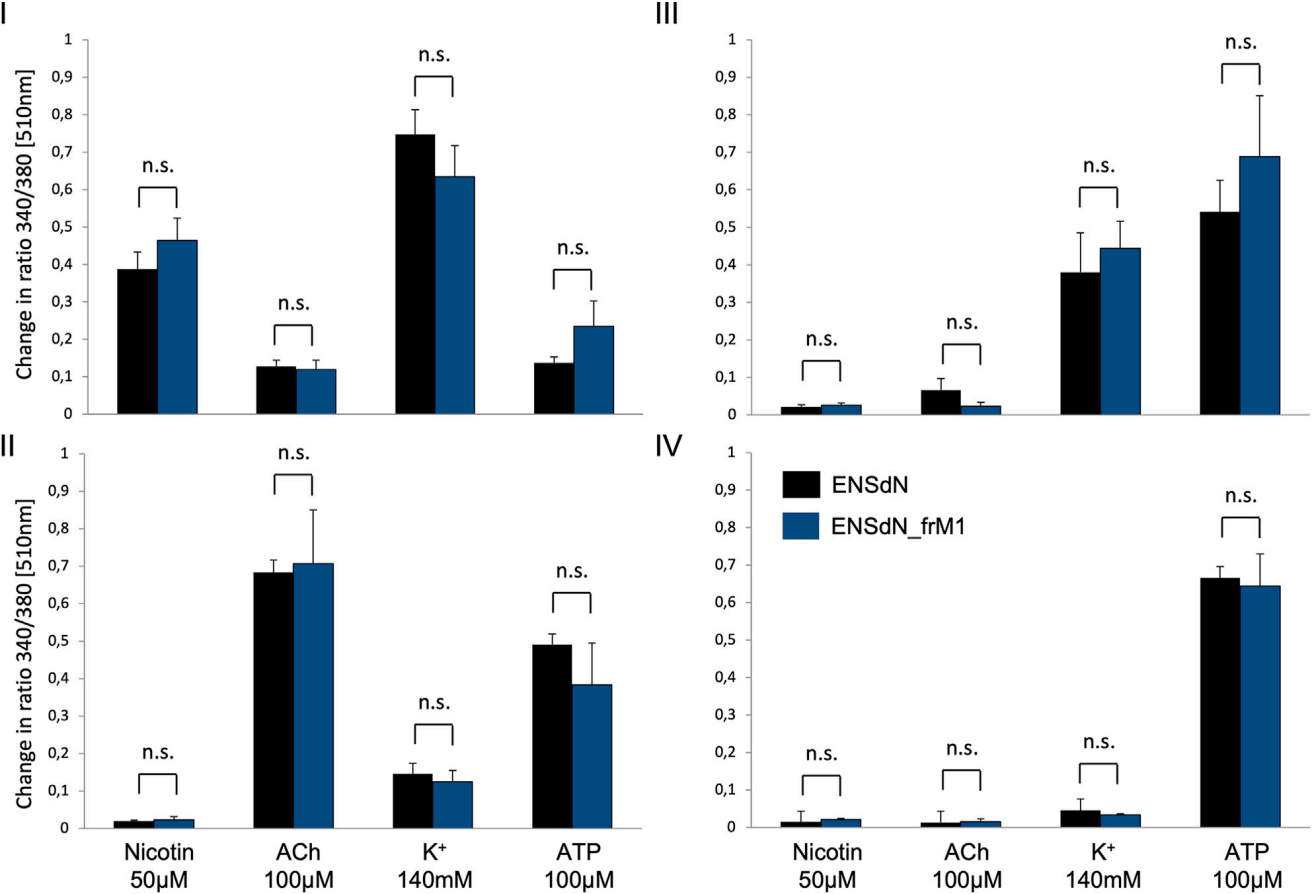
Mean change in fluorescence ratio in cells of functional group **(I–IV)** in ENSdN and ENSdN__frM1_. Mean change in ratio 340/380 nm [510 nm] of ENSdN (black) and ENSdN__frM1_ (blue) following subsequent stimulation with nicotine 50 μM, acetylcholine (ACh) 100 μM, K^+^ 140 mM and ATP 100 µM. Cells were assigned to subgroup **(I–IV)** according to response pattern (first rise in [Ca^2+^]_i_ after stimulation with **(I)** = nicotine, **(II)** = ACh, **(III)** = K^+^, **(IV)** = ATP); Student’s t-test ENSdN vs ENSdN__frM1_, n. s = not significant; data are shown as mean ± SEM; ENSdN n = 11; ENSdN__frM1_ n = 8.

Comparing the cells of functional subgroups I-IV, no significant difference could be observed for the mean transient rise of [Ca^2+^]_i_ (quantified as change in ratio 340/380 [510 nm]) for ENSdN and ENSdN__frM1_ (Figure 6).

Regarding the average number of cells in each group we could observe differences for ENSdN and ENSdN__frM1_. The percentage of cells in group I was 29.6 (±3.8%) for ENSdN and 48.7 (±4.8%) for ENSdN__frM1_—a difference that was considered significant (*p* = 0.007). A rise in [Ca^2+^]_i_ following stimulation with acetylcholine in cells that did not respond to a prior application of nicotine—representing cell group II—could be observed in 23.4 (±4.9%) cells for ENSdN and in 6.3 (±1.2%) cells for M1 group (*p* = 0.006).

**FIGURE 7:**
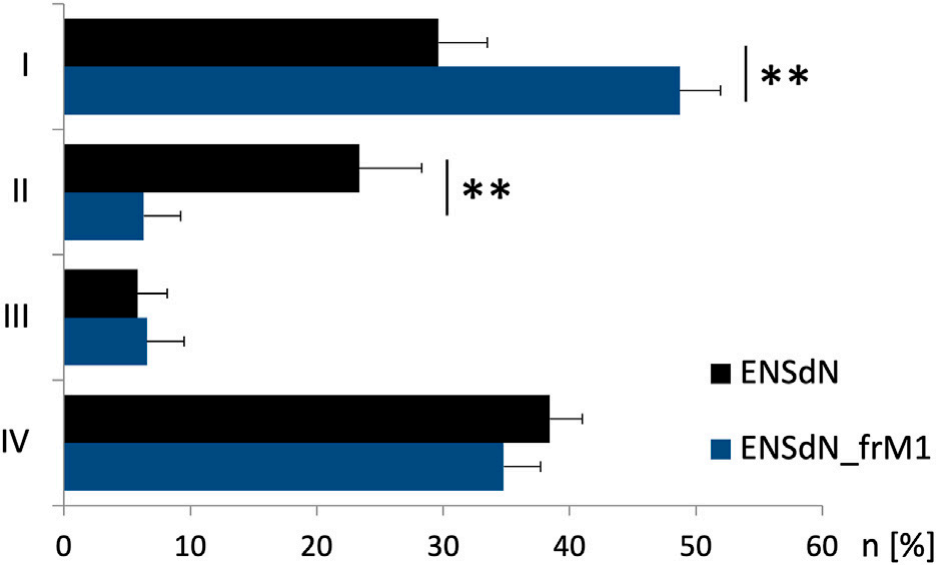
Number of cells of functional group **(I–IV)** before and after freezing. Cells were assigned to group **(I–IV)** according to response pattern (first rise in [Ca^2+^]_i_ after subsequent stimulation with nicotine 50 μM, acetylcholine (ACh) 100 μM, K^+^ 140 mM and ATP 100 µM (**(I)** = nicotine, **(II)** = ACh, **(III)** = K^+^, **(IV)** = ATP)). Mean percentage of cells/experiment assigned to subgroups I-IV for ENSdN or ENSdN__frM1_, Student’s t-test ENSdN vs ENSdN__frM1_.

No significant difference could be observed for the number of cells of group III and IV for ENSdN or ENSdN__frM1_ (Figure 7).

To summarize, freezing and thawing of ENSdN using condition M1 did not alter the increase in intracellular calcium in response to a specific set of stimuli. Single cells could be assigned to functional subgroups according to response patterns. A significant difference could be shown for the number of cells in subgroup I and II for frozen and unfrozen ENSdN.

## Discussion

Cryopreservation of cells is required when cells have to be stored for a certain period of time, e.g., in case of stem cell transplantation procedures. Storage conditions should ensure a sufficient survival rate of frozen cells without altering cell properties or interfering with cell function. Cryopreservation of various stem stells has been studied intensely over the last years. However, effects of cryopreservation on cell integrity and function can be varying for cells of different origin (Leibo and Mazur, 1971; Mazur et al., 2008), and we therefore particularly evaluated the influence of cryopreservation on enteric nervous system derived neurospheres (ENSdN). We tested three different slow freezing protocols containing the cryoprotectant DMSO, a substance that is known to increase pH (Morris, 2007). Cells in general are very sensitive to shifts of pH, and a DMSO-induced basic pH above 8.0 might be fatal for cell survival. In order to prevent damage caused by high pH in our setting we additionally used different concentrations of FCS to stabilize pH and protect the cells against freezing damage due to increased levels of albumin (Morris, 2007). Although DMSO is frequently used as cryoprotectant, it is cytotoxic and can induce cell differentiation (Adler et al., 2006). In retinal neuronal cell lines and rat hippocampal neurons DMSO induces cell death even in low concentrations of 0.5%–4% DMSO (Hanslick et al., 2009), whereas high concentrations of DMSO >10% exhibit toxicity via plasma membrane pore formation (Galvao et al., 2014). Moreover, in clinical settings of stem cell infusion it has been assumed that DMSO might cause toxic systemic reactions such as vomiting, cardiac dysfunction, and arrhythmia (Uemura and Ishiguro, 2015). Thus, DMSO-free or DMSO-diminished cryoprotective agents might be the better alternatives for ENSdN. Therefore we additionally used a DMSO free method (StemCellKeep™) based on a procedure called vitrification (Baharvand et al., 2010; Li et al., 2010; Hunt, 2011; Wagh et al., 2011; Pruksananonda et al., 2012; Beier et al., 2013), that has been successfully used for the storage of human ES/iPS cells (Matsumura et al., 2011). Carboxylated ɛ-poly-L-lysine (CPLL) is used as a cryoprotectant instead of DMSO, which is used widely as a food additive and is less cytotoxic than DMSO (Shima et al., 1984). However, in our experiments we did not achieve sufficient survival of intact or dissociated ENSdN using this method of cryopreservation. Additionally, gene expression was altered significantly in surving cells.

Concerning slow freezing methods, survival rates of human embryonic stem cells have been very low in the beginning (0.1%–1%). With specific modifications survival could be improved significantly (Ha et al., 2005). In current literature the survival rates of human embryonic and fetal neural progenitor cells are listed somewhere in between 20% and 40% (Nishiyama et al., 2016). By application of more sophisticated methods such as the combination of programmed freezing and magnetic field stimulation, survival could be increased up to 66% (Nishiyama et al., 2016). For adult stem cells, an even better survival rate has been shown (Karimi-Busheri et al., 2010; Martinello et al., 2011) and, for example, bulge-derived neural crest-derived stem cells from human hair follicles displayed survival rates of 82% (Gho et al., 2016).

Under the slow freezing conditions used in our study, we achieved survival rates between 27% and 40% for intact neurospheres and 45%–60% in dissociated cells, demonstrating that—in terms of cell survival—these methods seem to be an appropriate alternative to the cryopreservation of ENSdN. Supporting our observations, similar results have been shown by Imaizumi et al., who also observed higher survival rates for human pluripotent stem cells by slow freezing with DMSO (Imaizumi et al., 2014).

However, it has been reported that DMSO might initiate a coordinated differentiation program in various cell types (Jacob and Herschler, 1986; Morley and Whitfield, 1993). But obviously, the sensitivity of individual cell types to DMSO is prone to a huge variation (Laschinski et al., 1991). As a next step in our study, we therefore investigated the effect of DMSO (in freezing medium M1, M2 and M3) on mRNA expression of ENSdN__fr_. The quantity of mRNA of stem cell, neural and glial markers was analyzed by RT-PCR to assess changes in mRNA expression during the process of proliferation and as a second step after freezing of ENSdN. Interestingly, we observed a promotion of distinct progenitor, glial and neuronal markers during the process of cell proliferation, as well as alterations in mRNA expression profiles for the genes of interest after freezing with condition M1, M2 and M3.

Oct4 has been described as a specific marker for undifferentiated cells (Scholer, 1991; Pesce and Scholer, 2001) and Adler et al. found that DMSO has a untimely differentiation-inducing effect on embryonic cells (Adler et al., 2005). DMSO reduced the RNA expression of Oct4 in embryonic stem cells, indicating an effect of premature cell differentiation (Adler et al., 2006). However, in our setting we could observe rather an upregulation of Oct4 and other markers during proliferation and after freezing of cells, wich is in accordance to findings in murine neural precursor cells, where multipotency of cells was not affected by cryopreservation (Milosevic et al., 2005).

To evaluate the effect of freezing on differentiated cultures of ENSdN we assessed expression of neuronal, glial, and progenitor markers using immunohistochemical stainings.

Freezing of differentiated ENSdN with condition M1 (ENSdN__frM1_) did not alter protein expression at all in our setting. However, ENSdN__frM2_ and ENSdN__frM3_ resulted in an overexpression of Nanog, Oct4 and S100.

Since we achieved slightly better surviving rates in cultures of dissociated ENSdN, we additionally compared the protein expression of unfrozen differentiated spheres and dissociated cells (ENSdN and ENSdN__diss_). We could observe a significant lower expression of glial and stem cell markers (GFAP, Nestin and Sox2) in dissociated cells, suggesting an effect of the dissociation process on cell differentiation and expression patterns. However, the process of freezing did not additionally alter protein expression in dissociated cells (data not shown).

In addition to cell survival and alteration of protein and gene expression, we investigated the function of ENSdN and ENSdN__fr_. Since freezing cells using condition M1 did not significantly alter protein expression pattern in differentiated ENSdN, we chose ENSdN__frM1_ for functional imaging. Using calcium microfluorimetry with subsequent application of various stimuli, we could show no significant difference in the neuronal response to neurochemical stimuli (measured in level of rise in [Ca^2+^]_i_) of ENSdN and ENSdN__frM1_. We could identify different functional cell subgroups, that might match cell subtypes involved in ENS networks (Mann et al., 1999; Murphy et al., 2007; Mongardi Fantaguzzi et al., 2009), such as nicotinergic, cholinergic or non-cholinergic neurons, glial cells, smooth muscle cells or others. However, though the assignment of functional subgroups to a specific cell type seems tempting, unfortunately there is a lot of discussion about cell response patterns to different stimuli. In some studies, high potassium is used to distinguish neurons from glial cells, since glial cells do not display an acute Ca^2+^ transient upon K^+^depolarization (Gomes et al., 2009). In the complexity of ENS signaling, ATP seems to act as an important neurotransmitter that activates different receptors, e.g., P2Y. ATP in a concentration of 100 µM has been used to identify glial cells through the absence of neuronal calcium response (Kimball and Mulholland, 1996; Gulbransen and Sharkey, 2009; Fried and Gulbransen, 2015), whereas the effect was dependent on the concentration in other studies (Gomes et al., 2009), where glial cells predominantly responded to ATP (1 µM) while neurons needed concentration up to 5 µM of ATP for activation. Considering this, it can be assumed that the functional groups I-III in our study might match nicotinergic, cholinergic and non-nicotinergic/non-cholinergic neurons, whereas group IV is correlated with cells of glial origin.

The distribution of myenteric neurons has been investigated in different studies and is also dependent on species. For human colon, for example, 50% of neurons have been reported to contain choline acetyltransferase (ChAT) and 40% contain neuronal nitric oxide synthase (NOS) (Murphy et al., 2007), a rate that roughly matches our results.

It has been shown that the expression of neuronal or glial markers changes during different developmental stages of cells dependent on days of cultivation and/or the use of specific growth factors (Gomes et al., 2009), a fact that might also alter cell function. In this study we could observe a different distribution of functional cell types (possibly nicotinergic and cholinergic neurons) in differentiated unfrozen vs frozen cell cultures, which might be explained by different developmental stages when investigated on day 3 of cell culture.

## Conclusion

Cryopreservation of rodent ENSdN is possible and survival rates are sufficient to generate neurons and glial cells after freezing, but choosing the appropriate freezing mode and medium is crucial to prevent influence on cell gene and protein expression patterns. We believe that slow freezing of ENSdN with freezing condition M1 is the most appropriate method. It provided good cell survival, did not alter protein expression of differentiated spheres and did not impair neuronal function in live cell imaging. However, the distribution of neuronal cell subtypes might be affected by cryopreservation of cells.

## Figures and Tables

**Table:** 

**ENSdN**	Enteric Nervous System derived Neurospheres
**ENSdN**_**_diss**_	dissociated Enteric Nervous System derived Neurospheres
**ENSdN**_**_fr (M1, M2, M3, M4)**_	frozen Enteric Nervous System derived Neurospheres (treated with freezing and thawing condition M1, M2, M3 or M4)
**ENSdN**_**_fr (M1, M2, M3, M4) + diss**_	frozen and dissociated Enteric Nervous System derived Neurospheres (treated with condition M1, M2, M3 or M4)

## Data Availability

The raw data supporting the conclusion of this article will be made available by the authors, without undue reservation.
